# The Role of Lasers in Non-surgical Periodontal Treatment

**DOI:** 10.7759/cureus.102961

**Published:** 2026-02-04

**Authors:** Panteleimon Typou, Chariklia Neophytou, Konstantinos Papadimitriou

**Affiliations:** 1 Dentistry, School of Dentistry, Aristotle University of Thessaloniki, Thessaloniki, GRC; 2 Preventive Dentistry, Periodontology, and Implant Biology, Aristotle University of Thessaloniki, Thessaloniki, GRC; 3 Dentistry, School of Dentistry, European University Cyprus, Nicosia, CYP

**Keywords:** co2 laser, diode laser, er:yag laser, laser, nd:yag laser, non-surgical periodontal treatment

## Abstract

The use of lasers in non-surgical periodontal treatment has significantly increased, as they can contribute to the removal of subgingival calculus, disinfection and biomodification of the root surface, removal of diseased periodontal pocket epithelium, and antimicrobial activity against microflora. In addition, some types of lasers are proposed to promote the regeneration of periodontal tissues. This review aims to evaluate the role of lasers as an adjunct therapy in non-surgical periodontal treatment. A literature review was conducted, and systematic reviews and meta-analyses published in PubMed from 2020 to June 2025 were identified, with an emphasis on the clinical outcomes of laser treatment. A total of 10 systematic reviews/meta-analyses were obtained and assessed. Most findings indicate that the use of lasers as an adjunctive therapy provides statistically significant short-term benefits, such as a reduction in pocket depth and an improvement in clinical attachment level, compared with traditional scaling and root planing (SRP). However, long-term results (≥ 6 months) do not show a significant difference between the methods. Erbium lasers can be used as monotherapy, whereas diode and Nd:YAG (neodymium-doped yttrium-aluminum-garnet) lasers can be used as adjunctive therapy to SRP. The adjunctive use of lasers offers temporary clinical benefits to non-surgical periodontal treatment, but the long-term efficacy remains questionable. Further well-designed, multicenter clinical studies with standardized protocols and long-term follow-up are needed to document their efficacy.

## Introduction and background

The word LASER is an acronym for Light Amplification by Stimulated Emission of Radiation [1]. It was 1917 when Albert Einstein, through his Quantum Theory of Radiation, set the basis for the development of lasers. Albert Einstein’s theory stated the ability of stimulating segments of electromagnetic fields to generate intensified light [2]. The first working laser was constructed by Theodore Maiman at Hughes Research Laboratories in 1960 [3]. Since the 1980s, laser therapy has been introduced into periodontics, with the first reports documenting its application in periodontal surgery [1]. High-power lasers have gained various periodontal therapeutic indications after the incorporation of flexible optical fibers into their structure [3,4]. This evolution enabled the first clinical studies regarding the use of lasers in non-surgical periodontal treatment in the early 1990s, utilizing an Nd:YAG (neodymium-doped: yttrium-aluminum-garnet) laser [4].

Periodontitis is a common inflammatory disease that affects the periodontium (the gingiva, periodontal ligament, cementum, and alveolar bone) and progresses into a chronic condition [5,6]. This disease is more commonly observed in middle-aged and older adults [6], although it is not uncommon in children and adolescents [5]. In 2021, severe periodontitis affected over one billion individuals globally, with an age-standardized prevalence of 12.5% (10.53-14.49%). South Asia reported the highest regional prevalence at 17.57%. By 2050, the number of affected individuals is projected to exceed 1.5 billion, a 44.32% increase. Severe periodontitis remains a major public health concern, underlining the urgent need for effective, population-level prevention and management strategies [7].

Periodontitis has a multifactorial etiology, involving primarily a complex interplay between subgingival bacterial biofilm, particularly Gram-negative anaerobic periodontal pathogens, such as *Porphyromonas gingivalis, Treponema denticola, Tannerella forsythia, *and *Aggregatibacter actinomycetemcomitans*, and the host's immune-inflammatory response to this biofilm [6,8,9]. This host response can be modified by genetic predispositions, behavioral habits, and systemic conditions [9,10]. Although periodontal diseases are initiated by bacteria that colonize the tooth surface and gingival sulcus, the host response plays an essential role in the breakdown of connective tissue and bone, key features of the disease process [11].

The pathogenesis of periodontitis is initiated by the accumulation of bacterial biofilm in the subgingival region [8], which triggers the activation of innate immune cells (neutrophils and macrophages) and adaptive immune cells (lymphocyte T-cells and B-cells) [12], the production of pro-inflammatory cytokines (interleukin 1-beta (IL-1β), tumor necrosis factor-alpha (TNF-α), and interleukin-6 (IL-6)) amplifying the inflammatory response [11], and the secretion of matrix metalloproteinases (MMPs), particularly MMP-8 and MMP-9, by neutrophils and fibroblasts, resulting in collagen breakdown in the periodontal ligament and gingival connective tissue [13]. Simultaneously, inflammatory cytokines stimulate the expression of RANKL (Receptor Activator of Nuclear factor Kappa-B Ligand), which promotes the formation and activation of osteoclasts, leading to resorption of alveolar bone [11]. The junctional epithelium migrates apically because of the loss of connective tissue support, forming a periodontal pocket, a hallmark of periodontitis [6]. A periodontal pocket is essentially a pathologically deepened gingival sulcus that is caused by the destruction of the connective tissue attachment and the resorption of alveolar bone due to chronic inflammation [14]. Periodontitis is characterized by progressive destruction of periodontal tissues, loss of clinical attachment and alveolar bone, and formation of periodontal pockets, and if left untreated, it can lead to tooth mobility and tooth loss [6].

The primary goal of periodontal therapy remains the preservation of natural dentition in a state of health, comfort, esthetics, and function for as long as possible [15]. Scaling and root planing (SRP) is a basic mechanical procedure in the treatment of periodontitis that aims at the removal of the supra- and subgingival biofilm and calculus from colonized root surfaces [9]. SRP can resolve inflammation and heal the periodontal pockets, mainly by the formation of a long epithelial junction. SRP is a well-documented technique and is considered the “gold standard” therapy in the treatment of periodontitis [8]. However, SRP has limitations, especially in anatomically complex areas, such as furcations, grooves, and deep periodontal pockets. Moreover, mechanical instrumentation may not always eliminate all bacterial endotoxins from root surfaces [16]. An additional challenge in the management of periodontitis is the growing resistance of subgingival microorganisms to antimicrobials administered either locally or systemically as adjuncts to SRP. This resistance, along with other limitations of conventional therapies, has underlined the need for alternative approaches in non-surgical periodontal treatment. Among these, the use of lasers has been explored for several years as an adjunctive modality to standard periodontal procedures. Nevertheless, the efficacy of laser therapy in this context remains a subject of ongoing debate and clinical investigation [8].

The utilization of laser devices in periodontology has emerged as an alternative or adjunctive approach to conventional periodontal therapy [16]. Lasers offer a wide spectrum of clinical applications, ranging from non-surgical periodontal treatment to surgical periodontal treatment and periodontal regeneration [16,17]. The therapeutic effectiveness of lasers is based on their photothermal interaction with biological tissues and their bactericidal properties at temperatures above 50°C. As Gram-negative anaerobic bacteria absorb the irradiation emitted by the different types of lasers (Nd:YAG and diode), they are destroyed, leading to the reduction of inflammation and the enhancing of tissue healing [16]. Additionally, lasers support microorganisms’ and tissue’s ablation or vaporization, hemostasis, coagulation, and biostimulation, and promote healing [8,16].

Lasers can be used in non-surgical periodontal treatment, enhancing the outcomes of the conventional SRP [4] and even promoting the regeneration of periodontal tissues [16]. The purpose of this study is to investigate the role of lasers in non-surgical periodontal treatment.

## Review

Materials and methods

This review was conducted as a narrative synthesis of the available literature regarding the application of lasers in non-surgical periodontal treatment. This study was focused on evaluating systematic reviews and meta-analyses that assessed the adjunctive or alternative use of various types of lasers compared to conventional SRP.

Literature Sources and Eligibility

We included systematic reviews and meta-analyses published in the PubMed electronic database between 2020 and June 2025. The literature search was conducted using PubMed, selected for its comprehensive coverage of peer-reviewed medical and dental journals. In addition, several of the included systematic reviews and meta-analyses had already incorporated studies retrieved from other databases such as Scopus and the Cochrane Library, thereby indirectly broadening the evidence base. The systematic review by Coluzzi et al. [10] is the only systematic review that is based exclusively on the PubMed database. Given the narrative design of the present review and its focus on high-level evidence, no separate searches of additional databases were performed. The literature search was conducted in PubMed using combinations of the following keywords: (‘laser’ OR ‘laser therapy’) AND (‘non-surgical periodontal treatment’). Filters were applied for human studies, English language, and systematic reviews or meta-analyses published between January 2020 and June 2025. Inclusion criteria included: (1) human in vivo studies, (2) adult patients diagnosed with periodontitis, including those with systemic conditions such as diabetes mellitus, (3) use of any type of lasers (Er:YAG - Erbium-doped: yttrium-aluminum-garnet, Er,Cr:YSGG - erbium, chromium-doped: yttrium-scandium-gallium-garnet, Nd:YAG, diode, KTP - potassium titanyl phosphate, CO_2_ - carbon dioxide, or low-level lasers) as monotherapy or as adjunctive therapy to SRP, (4) comparisons made with SRP alone or with placebo interventions, (5) reported clinical outcomes, such as probing depth (PD), clinical attachment level (CAL), bleeding on probing (BoP), gingival index (GI), plaque index (PI), gingival recession (GR), and patient-reported outcomes (e.g., pain via visual analogue scale (VAS)), and (6) minimum follow-up period of one month.

Exclusion criteria included animal studies, in vitro studies, narrative reviews without systematic methodology, case reports, and studies lacking clear clinical endpoints. It should also be acknowledged that very recent individual randomized controlled trials (RCTs) or ongoing clinical studies published outside the indexing period or databases used may not have been captured.

Data Extraction and Outcomes

From each study, the following data were extracted: study design (systematic review, meta-analysis), sample size, patient characteristics (e.g., systemically healthy, smokers, diabetes mellitus), type of laser, wavelength, power settings, irradiation protocols, and whether lasers were applied as monotherapy or adjunctive therapy, duration of follow-up (ranging from short-term (1-3 months) to long-term (≥12 months)), primary outcomes: changes in PD and CAL, and secondary outcomes: BoP, PI, GI, GR, microbiological changes, inflammatory markers (e.g., IL-1β, TNF-α), metabolic parameters (glycated hemoglobin HbA1c in diabetic patients), and patient-related outcomes (VAS for pain).

Quality and Heterogeneity Assessment

Study limitations, risk of bias, and methodological heterogeneity were recorded, with particular attention to inconsistent laser parameters (power, energy density, angulation, irradiation mode), variability in SRP protocols across studies, follow-up duration differences (short- vs. long-term), inclusion/exclusion of smokers and patients with systemic diseases, and lack of standardized outcome measurements and inadequate reporting of laser protocols. A formal, standardized risk-of-bias tool (e.g., AMSTAR 2) was not applied, which represents a limitation of the present review.

Synthesis Approach

Due to the heterogeneity of laser types, clinical protocols, and outcome measures, a descriptive synthesis approach was only applied. Where available, meta-analytic data were highlighted to present pooled estimates of PD and CAL changes. In addition, subgroup findings were described, such as effects observed in smokers, patients with diabetes, and those treated with combined laser modalities (e.g., Er:YAG + Nd:YAG).

Results

A total of 10 systematic reviews/meta-analyses were obtained and assessed [10,18-26]. The purpose of this study is to investigate the role of lasers in non-surgical periodontal treatment.

A systematic review of 20 RCTs using Er:YAG, Er,Cr:YSGG, Nd:YAG, diode, and KTP lasers reported improvements in PD and CAL when lasers were used adjunctively to SRP. Approximately 70% of studies found significant benefits in at least one key parameter (PD, CAL, BoP), while 30% showed no additional advantage. Improvements in inflammatory biomarkers, such as IL-1β and TNF-α, were also observed. Follow-up periods varied from 6 to 20 months; not all were long enough for reliable evaluation of long-term outcomes [10].

A systematic review of 16 RCTs (525 patients) evaluating the adjunctive use of lasers (Er:YAG, Er,Cr:YSGG, Nd:YAG, diode, CO_2_, and low-level lasers) showed that SRP, combined with laser therapy, significantly improved PD and CAL at one, three, and six months compared to SRP alone. Additional reductions were observed in PI and GI except at 24 weeks, when PI was not significantly different. Gingival crevicular fluid (GCF) volume decreased, but cytokine levels (IL-1β, IL-6) showed no consistent differences. Importantly, benefits were greater among non-smokers, whereas smokers exhibited reduced or non-significant improvements. No adverse events were reported, suggesting the safety of lasers as adjuncts [18].

A meta-analysis of 16 RCTs (606 patients) demonstrated that Er,Cr:YSGG lasers, used either as adjuncts or substitutes to SRP, provided significant short-term improvements in PD reduction (WMD = −0.35 mm, 95% CI (−0.63, −0.07), P = 0.013 at one month; WMD = −0.342 mm, 95% CI (−0.552, −0.132), P = 0.001 at three months) and CAL gain (WMD = −0.17 mm, 95% CI (−0.31, −0.03), P = 0.017 at three months). Additionally, patients reported lower postoperative pain (VAS score WMD = −2.395, 95% CI (−3.327, −1.464), P = 0.000). However, these benefits were not sustained at six months, indicating limited long-term efficacy [19].

Eight RCTs/controlled clinical trials (CCTs) comparing laser monotherapy (Er:YAG, diode, Nd:YAG) to mechanical instrumentation showed no statistically significant differences in PD (WMD = 0.14 mm; 95% CI (−0.04, 0.32); z = 1.51; p = 0.132) or CAL (WMD = 0.04 mm; 95% CI (−0.35, 0.42); z = 0.19; p = 0.850). This confirms that conventional mechanical debridement remains the standard of care in untreated periodontitis patients [20].

An umbrella review of four systematic reviews concluded that Er:YAG lasers, whether as adjuncts or alternatives to SRP, did not demonstrate significant long-term effects on PD, CAL, GR, or PI. Short-term improvements in PD reduction and reduced patient pain were observed, but these did not persist beyond three months [21].

A systematic review of 15 RCTs on diode lasers (808-980 nm) as adjuncts to SRP reported high variability and inconsistencies in clinical outcomes. Some studies demonstrated added value for PD and CAL reduction in the short term, but overall results were heterogeneous and inconclusive. Incomplete reporting of laser parameters (~50% missing details, such as tip initiation, power density, spot size) further limited comparability. Most studies had small sample sizes (<50 patients), short follow-up periods (≤6 months), and high to moderate risk of bias. Nevertheless, adjunctive diode lasers appeared to provide some clinical benefit, warranting further multicenter, well-designed RCTs with standardized protocols [22].

In diabetic patients with periodontitis, adjunctive diode laser therapy demonstrated significant short-term improvements in PD and CAL, along with modest HbA1c reductions at 3-6 months [23,24]. Corbella et al. [23] reported greater PD reduction (MD = 0.59 mm, 95% CI (0.41, 0.76), I^2^ = 80%, 170 subjects) and CAL gain (MD = 0.84 mm, 95% CI (0.09, 1.59), I^2^ = 86%, 112 subjects) at three months when diode laser is used in conjunction with SRP. Similarly, Zhao et al. [24] demonstrated significant improvements in PD reduction (MD = 0.47 mm; 95% CI (0.21 to 0.74), P < 0.01 after three months and MD = 0.32 mm; 95% CI (0.17 to 0.48), P < 0.01 after six months) and CAL gain (MD = 0.37 mm; 95% CI (0.16 to 0.58), P < 0.05 after three months and MD = 0.26 mm; 95% CI (0.05 to 0.48), P < 0.05 after six months) at both three and six months, with no significant differences observed at one month. According to Corbella et al. [23], in terms of glycemic control, adjunctive diode laser therapy resulted in modest but statistically significant reductions in HbA1c at three months (MD = 0.18%, 95% CI (0.07, 0.28)). Similarly, Zhao et al. [24] reported that adjunctive use of diode laser therapy to SRP led to significantly greater reductions in HbA1c at three (MD = 0.19%; 95% CI (0.10 to 0.28)) and six months (MD = 0.22%; 95% CI (0.03 to 0.41)) compared to SRP alone. However, heterogeneity in patient selection, glycemic control, and laser protocols limited generalizability, and current evidence remains insufficient to recommend routine use in this population [23,24].

Limited RCTs investigating combined Nd:YAG and Er:YAG therapy suggested superior PD reduction (MD = 1.08 mm; 95% CI (0.24 to 1.91), P = 0.01) and CAL gain (MD = 0.95 mm; 95% CI (0.63 to 1.28), P < 0.00001) compared to SRP alone. However, these studies were few, short-term (≤6 months), and heterogeneous in laser settings [25].

A synthesis of 15 systematic reviews concluded that erbium lasers (Er:YAG, Er,Cr:YSGG), when used as monotherapy or adjunct to SRP, provided better short-term outcomes for PD, CAL, and BoP, but results were comparable to SRP alone at ≥6 months. Patient-reported outcomes such as pain relief favored laser-assisted therapy, although evidence was limited [26].

Discussion

One of the major challenges in the treatment of periodontal disease is the complete removal of plaque and calculus and the achievement of a clean and smooth root surface that prevents plaque and calculus retention and promotes good gingival reattachment [17]. Lasers can be used in non-surgical periodontal treatment for the removal of subgingival calculus, the modification of root surface, the bactericidal effects and detoxification of root surface, the removal of the diseased pocket wall, and the antimicrobial effect on the pocket microflora [4]. It has been suggested that certain types of lasers can also promote the regeneration of periodontal tissues [16].

CO_2_ Laser

The CO_2_ laser at high energy and continuous wave settings is not suitable for SRP [2], because it can easily cause melting and carbonization of calculus [4], thermal effects on pulp function [27], and undesirable alterations on the root surface (craters, pores, melting and carbonization of cementum) [4,27]. Conversely, when used at low energies and pulsed or defocused, CO_2_ laser could be used in combination with mechanical non-surgical periodontal therapy to create a smooth root surface, accelerating pocket healing [28], to reduce the microbial load [2], and to seal dentinal tubules, improving fibroblast adhesion [28] without the risk of causing thermal side effects to the tissues [2].

However, its clinical efficacy remains limited, as the CO_2_ laser is less effective than ultrasonic devices and the Nd:YAG laser in the removal of subgingival plaque and calculus [28]. Pope et al. observed that areas treated with CO_2_ laser in combination with mechanical non-surgical treatment tended to have greater reduction in PD, greater recession, and greater gains in CAL, without statistically significant difference compared to the mechanical SRP when applied alone [29].

Nd:YAG Laser

The Nd:YAG laser cannot completely remove subgingival calculus, even at clinically appropriate energy parameters [2]. An ideal setting for the use of the Nd:YAG laser is 100 mJ/pulse, in order to avoid the risk of increasing intrapulpal temperature [2], to avoid changes in the root surface (craters, cavities, pores and melting, carbonation and decarbonation of cementum) [27], and to avoid the reduced effectiveness of low energy settings, providing possible benefits in microbial disinfection, with uncertain long-term effectiveness [2]. The Nd:YAG laser can target inflamed periodontal tissue and selectively remove it compared to the diode laser [30].

The Nd:YAG laser is recommended to be used adjunctively, specifically following the initial mechanical non-surgical periodontal treatment [2]. Adjunctive treatment with Nd:YAG laser has demonstrated several biological benefits, including reduction of subgingival microbes (*A. actinomycetemcomitans, P. gingivalis, P. intermedia, B. subtilis,* and *E. coli*) [27] and inactivation of endotoxins in periodontally diseased roots [4]. Moreover, a reduction of ICAM-1 (endothelium intercellular adhesion molecule-1), IL-1β, and TNF-α levels in the gingival crevicular fluid (GCF) is observed, particularly at three- and six-month follow-up, thus inhibiting the inflammatory process and alleviating periodontitis [30,31]. Additionally, Nd:YAG laser application has been associated with increased total antioxidant capacity of GCF [32].

Adjunctive treatment with Nd:YAG laser does not seem to further improve periodontal PD measurements and CAL when compared to SRP alone. The use of Nd:YAG laser led to similar results as conservative mechanical periodontal treatment in reducing CAL and PD and has no additional advantage in locations with PD 4-6.5 mm, with ongoing debate about its superiority. Long-term studies with larger sample sizes are needed, focusing on the treatment of deeper pockets greater than 4-6.5 mm [2].

The Nd:YAG laser is the only laser that has been reported to achieve histological results of tissue regeneration and new attachment [30]. However, there are no CCSs, and the controlled study by Yukna et al. did not have a large sample of teeth [30,33]. In patients with moderate to severe periodontitis, Nd:YAG laser treatment combined with careful occlusion adjustment, SRP, antibiotic use, and other factors included in the LANAP (laser-assisted new attachment procedure) protocol has reported encouraging clinical and histological results. Nd:YAG laser adjusted appropriately to remove surgically diseased epithelium, to stabilize the clot, and to eliminate pathogenic microorganisms appeared to create an environment suitable for periodontal regeneration or new attachment mediated by cementum. Although regenerative results have been reported through other surgical techniques, laser treatment is associated with fewer complications and lower morbidity [30].

A systematic review used a combined protocol of Er:YAG and Nd:YAG, which have been suggested to provide synergistic effects, particularly in deep pockets, by combining Er:YAG’s calculus removal ability with Nd:YAG’s antimicrobial, coagulative effects, and the ability to remove the sulcular epithelium. This approach appears to achieve superior PD reduction (1.08 mm) and CAL gain (0.95 mm) compared with SRP alone. However, these studies were few, short-term (≤6 months), and heterogeneous in laser settings [25].

Erbium Lasers (Er:YAG and Er,Cr:YSGG Lasers)

Erbium lasers can be used either as an alternative or a complementary treatment to the mechanical non-surgical periodontal treatment [2]. When erbium lasers are set at 160 mJ and at a frequency of 10 Hz, they can effectively remove subgingival calculus and necrotic cementum [34], improve clinical parameters, such as PD, CAL and BoP [2], reduce the levels of subgingival microbes (*A. actinomycetemcomitans, P. gingivalis, P. intermedia, T. forsythia, T. denticola, F. nucleatum,* anaerobes), controlling their growth [2,27], remove bacterial endotoxins and lipopolysaccharides (LPS) from root surfaces [34] and reduce the levels of IL-1β and TNF-α in the GCF [20].

Erbium lasers, properly adjusted, did not reveal clinical residual calculus or showed smaller amounts of calculus compared to conventional instruments. The ability of the Er:YAG laser to eliminate calculus is comparable to ultrasound and hand instruments, while its effectiveness appeared to be much greater compared to the Er,Cr:YSGG laser. The root surface treated with the erbium laser was relatively rougher than that of SRP, without significant thermal damage, and with an increase in the amount of cementum removed [17]. The Er:YAG laser is considered to be the most effective laser in non-surgical periodontal treatment for the removal of subgingival calculus, causing minimal damage to adjacent periodontal tissues without overheating the tooth pulp, when adjusted to the appropriate energy conditions [20].

Erbium lasers applied at appropriate power levels (160 mJ/pulse) can provide clear advantages in terms of PD, CAL, and BoP. Erbium laser treatment was more time-efficient compared to traditional non-surgical periodontal therapy. The effectiveness of erbium lasers in the treatment of moderate (4-6 mm) and deeper pockets (7 mm or more) is proven in the long term, while in areas such as root furcations, it remains uncertain in the long term [2]. According to Lin et al., in patients with untreated periodontitis, Er:YAG laser monotherapy did not show superior clinical benefits compared to conventional periodontal treatment [20].

The adjunctive use of laser Er,Cr:YSGG can lead to similar CAL gains to SRP alone with less recession, statistically significant reduction in PD [35], and clinical reduction in inflammation in the first three months [36]. Further research should be conducted on the adjunctive use of Er,Cr:YSGG in conventional non-surgical periodontal therapy [35].

Systematic reviews analyzed in the present study indicate the short-term improvements in PD and CAL when erbium lasers are used adjunctively or alternatively to SRP, though long-term benefits beyond six months are not consistently supported [19,26]. Meta-analyses suggest that erbium lasers improve PD and CAL reduction in the short term but show no clear superiority to SRP at ≥6 months [26].

Diode Lasers

Diode lasers with wavelengths of 630-690 nm, as well as 805 nm and 808-810 nm, are predominantly used for antibacterial photodynamic therapy (aPDT). Lasers operating at 940 nm and 980 nm are primarily indicated for bacterial decontamination, soft-tissue curettage in periodontal pockets, and photobiomodulation therapy [8]. When applied as adjuncts to SRP, diode lasers demonstrate notable anti-inflammatory and bactericidal effects [22]. Systematic reviews report short-term improvements in PD and CAL -particularly in patients with diabetes, where adjunctive diode laser therapy has also been associated with modest reductions in HbA1c levels [23,24]. However, inconsistencies across trials, lack of standardized protocols, and variability in microbiological outcomes continue to limit definitive conclusions [22].

General Findings Across Studies

Overall, adjunctive laser therapy to SRP consistently showed short-term improvements in PD and CAL, particularly in non-smokers and in patients with systemic conditions such as diabetes [10,18-21,23-26]. However, long-term benefits remain unproven, with substantial heterogeneity in study design, laser protocols, and follow-up durations [10,18,20-22,24,26]. Microbiological and biochemical outcomes were inconsistent, and only a few studies have yet incorporated patient-reported measures or economic evaluations [20-22,26].

General Considerations

Across different laser systems, certain consistent issues can be identified. Evidence frequently demonstrates short-term improvements in PD reduction and CAL gain, and decreases in BoP. However, the persistence of these benefits beyond six months has not been conclusively established [10,18,26]. In addition, considerable heterogeneity is observed among studies with respect to laser type, wavelength, energy parameters, and patient characteristics, which complicates direct comparisons and limits the robustness of meta-analytic conclusions [20,22].

## Conclusions

Overall, adjunctive laser therapy to SRP consistently showed short-term improvements in PD and CAL, particularly in non-smokers and in patients with systemic conditions such as diabetes. However, long-term benefits remain unproven. While lasers cannot yet be considered replacements for SRP, their adjunctive use may enhance treatment outcomes in selected patient groups, particularly those with deep pockets, inaccessible sites, or systemic conditions such as diabetes. Future research should prioritize standardizing laser protocols, conducting larger, longer-term RCTs, and evaluating patient-reported outcome measures and cost-effectiveness.
